# Biglycan Involvement in Heart Fibrosis: Modulation of Adenosine 2A Receptor Improves Damage in Immortalized Cardiac Fibroblasts

**DOI:** 10.3390/ijms24021784

**Published:** 2023-01-16

**Authors:** Michele Scuruchi, Federica Mannino, Chiara Imbesi, Giovanni Pallio, Giovanna Vermiglio, Gianluca Bagnato, Letteria Minutoli, Alessandra Bitto, Francesco Squadrito, Natasha Irrera

**Affiliations:** 1 Department of Clinical and Experimental Medicine, University of Messina, Via C. Valeria, 98125 Messina, Italy; 2 Department of Biomedical, Dental, Morphological and Functional Imaging Sciences, University of Messina, Via C. Valeria, 98125 Messina, Italy

**Keywords:** cardiac fibrosis, A_2A_ receptor, biglycan, collagen

## Abstract

Cardiac fibrosis is a common pathological feature of different cardiovascular diseases, characterized by the aberrant deposition of extracellular matrix (ECM) proteins in the cardiac interstitium, myofibroblast differentiation and increased fibrillar collagen deposition stimulated by transforming growth factor (TGF)-β activation. Biglycan (BGN), a small leucine-rich proteoglycan (SLRPG) integrated within the ECM, plays a key role in matrix assembly and the phenotypic control of cardiac fibroblasts. Moreover, BGN is critically involved in pathological cardiac remodeling through TGF-β binding, thus causing myofibroblast differentiation and proliferation. Adenosine receptors (ARs), and in particular A_2AR_, may play a key role in stimulating fibrotic damage through collagen production/deposition, as a consequence of cyclic AMP (cAMP) and AKT activation. For this reason, A_2AR_ modulation could be a useful tool to manage cardiac fibrosis in order to reduce fibrotic scar deposition in heart tissue. Therefore, the aim of the present study was to investigate the possible crosstalk between A_2AR_ and BGN modulation in an in vitro model of TGF-β-induced fibrosis. Immortalized human cardiac fibroblasts (IM-HCF) were stimulated with TGF-β at the concentration of 10 ng/mL for 24 h to induce a fibrotic phenotype. After applying the TGF-β stimulus, cells were treated with two different A_2AR_ antagonists, Istradefylline and ZM241385, for an additional 24 h, at the concentration of 10 µM and 1 µM, respectively. Both A_2AR_ antagonists were able to regulate the oxidative stress induced by TGF-β through intracellular reactive oxygen species (ROS) reduction in IM-HCFs. Moreover, collagen1a1, MMPs 3/9, BGN, caspase-1 and IL-1β gene expression was markedly decreased following A_2AR_ antagonist treatment in TGF-β-challenged human fibroblasts. The results obtained for collagen1a1, SMAD3, α-SMA and BGN were also confirmed when protein expression was evaluated; phospho-Akt protein levels were also reduced following Istradefylline and ZM241385 use, thus suggesting that collagen production involves AKT recruited by the A_2AR_. These results suggest that A_2AR_ modulation might be an effective therapeutic option to reduce the fibrotic processes involved in heart pathological remodeling.

## 1. Introduction

Cardiac fibrosis consists of the aberrant deposition of extracellular matrix (ECM) proteins in the cardiac interstitium, which mostly occurs following myocardial infarction, hypertensive heart diseases and cardiomyopathies and significantly contributes to heart failure [1]. As some cells responsible for ECM turnover, cardiac fibroblasts (CFs) are recognized as one of the main effectors in the onset of fibrotic scar deposition in the heart [2,3]. These cells are mainly involved in ECM production and turnover in order to maintain heart homeostasis; however, when an injury occurs, their aberrant activation and differentiation into myofibroblasts is associated with impaired ECM deposition, thus inducing fibrotic scar formation with the consequent replacement of the normal tissue with the fibrotic one. This altered heart architecture impairs cardiac function with significant consequences in the clinical setting, in terms of cardiovascular risk, and worsens patient outcomes [4].

Cardiac fibrosis is a complex process characterized by the involvement of different cell types, cytokines, chemokines and growth factors, including transforming growth factor (TGF)-β, of which activation induces myofibroblast differentiation and stimulates type I collagen and connective tissue growth factor (CTGF) release, thus worsening the quality of the scar. For this reason, TGF-β is considered a negative predictive factor for heart failure [5].

TGF-β plays important roles in fibroblast functions, including proliferation, cytokine secretion, cytoskeletal rearrangements and ECM deposition and remodeling [6,7,8,9]. TGF-β may be the trigger of the NLRP3 inflammasome of which activation not only contributes to the increase in ROS production but also to IL-1β and caspase-1 release [10,11,12,13].

Inflammasome associated-molecules, such as TGF-β and IL-1β, are critically involved in the onset and progression of fibrosis, thus activating fibroblasts and inducing ECM deposition [14]. Matrix production may also be regulated by adenosine A_2A_ receptors through cyclic AMP release and activation of its downstream signaling molecules. In fact, fibrosis may occur via A_2A_-mediated cAMP/PKA signaling [15], and the use of caffeine as a nonselective adenosine receptor antagonist has been observed to be protective against liver fibrosis through the modulation of this pathway.

In this setting, previous studies have shown cross-talk among adenosine receptors, TGF-β, and AKT signaling pathways in would healing and fibrosis, underlying the importance of pharmacological targeting of adenosine receptors in the control of fibrotic mechanisms [16,17].

The regulatory actions of adenosine receptors engage different interconnected pathways, and several data also described biglycan (BGN)/TGF-β axis in fibrosis and in the pathological remodeling of the heart. BGN binds TGF-β, thus driving myofibroblast differentiation and proliferation through TGF-β activation [18,19,20]; in fact, increased TGF-β activity has been identified as a positive regulator of BGN expression [21,22] and the BGN/TGF-β axis is involved in the pathological remodeling of the heart, including fibrotic scar formation [20].

In particular, BGN is a small leucine-rich proteoglycan (SLRPG), belonging to a family of proteoglycans (PGs), characterized by a central protein core with leucine-rich motifs and one or two chondroitin/dermatan sulphate glycosaminoglycan (GAG) chain(s) near the N-terminal end [23]. This PG is integrated within the ECM or in the pericellular space and plays a key role in matrix assembly and is involved in a plethora of cell functions, such as cell growth and migration, apoptosis, inflammation and fibrosis [24,25].

In the context of cardiac pathophysiology, BGN expression is upregulated during healing processes and drives phenotypic changes of cardiac fibroblasts [26]. Additionally, BGN deficiency attenuates fibrotic scar deposition and preserves cardiac function during pressure overload-induced cardiac remodeling in mice [27]. The protective role of BGN downregulation is probably due to its involvement as a structural molecule in the assembly of collagen, as well as its action as a signaling molecule in stimulating different cell functions [20,24,25]. In fact, previous studies already demonstrated that the adenosine A_2A_ receptor may contribute to the development of fibrotic damage mainly through the stimulation of collagen production and deposition [28,29], and its modulation might be useful to manage cardiac fibrosis by limiting fibrotic scar deposition in tissue [30] and also in the heart [31,32].

In light of this background, the aim of the present study was to investigate the possible crosstalk between the A_2AR_ and BGN pathways using an in vitro model of TGF-β-induced fibrosis. 

## 2. Results

### 2.1. Treatment with Istradefylline and ZM241385, Two Different A_2A_ Receptor Antagonists, Does Not Affect IM-HCF Cell Viability 

**Figure 1:**
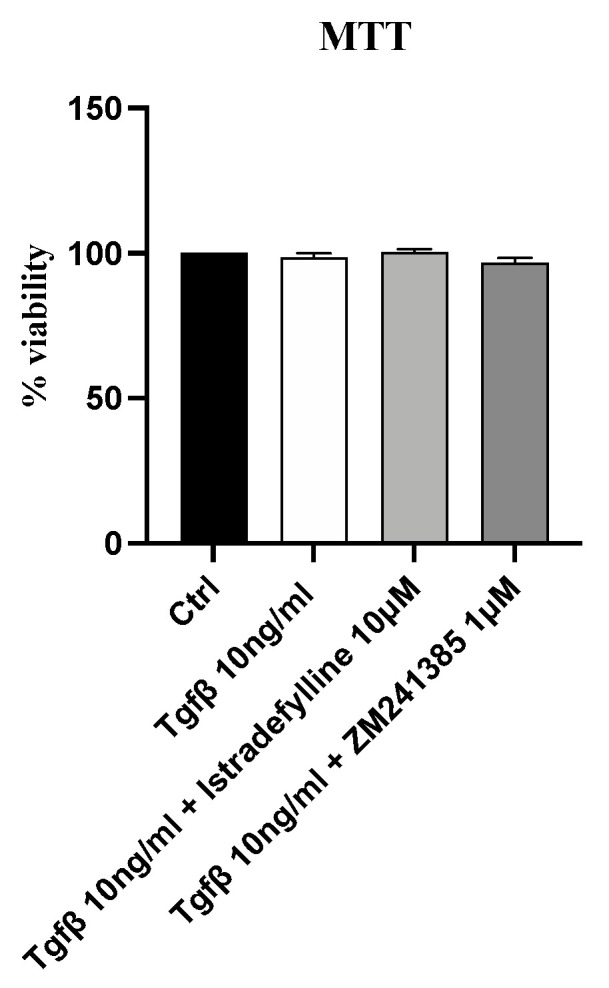
Cytotoxicity assay evaluated with MTT in the IM-HCF cell line stimulated with TGF-β (10 ng/mL) alone and TGF-β followed by Istradefylline (10 µM) or ZM241385 (1 µM) for 24 h. Values are expressed as the means and SD of five experiments.

In order to evaluate whether Istradefylline or ZM241385 could have cytotoxic effects, cell viability was assessed after incubating IM-HCF cells with TGF-β alone and following Istradefylline or ZM241385 treatment for 24 h. TGF-β stimulation did not reduce cell viability at the concentration of 10 ng/mL; even the incubation with TGF-β plus Istradefylline or ZM241385 did not affect cell viability of IM-HCF cells after 24 h of exposure (Figure 1), thus indicating that the concentrations of both the pro-fibrotic stimulus and treatments were not toxic for cells. 

### 2.2. Antagonism of A_2AR_ Reduces Intracellular ROS Production

**Figure 2:**
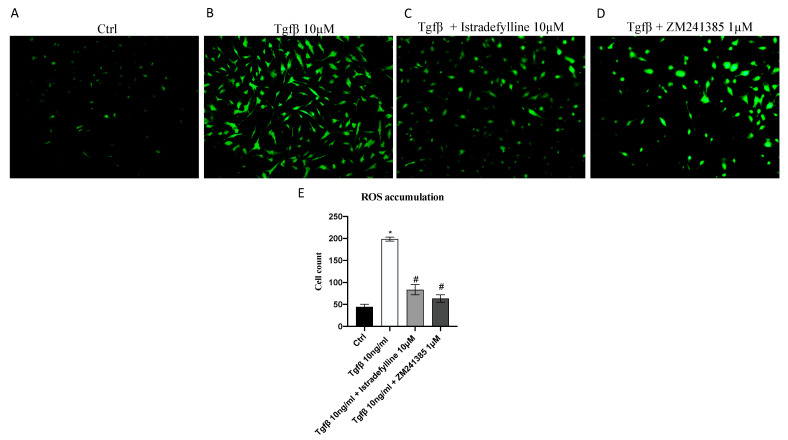
Intracellular ROS accumulation evaluated with a CM-H2DCFDA fluorescent probe in IM-HCF cells stimulated with TGF-β (10 ng/mL) alone and TGF-β followed by Istradefylline (10 µM) or ZM241385 (1 µM) for 24 h. Panel E shows the % of fluorescent cells/total cells. The data are expressed as means ± SDs of five experiments. * *p* < 0.05 vs. CTRL; # *p* < 0.05 vs. TGF-β. All images were captured at 10× magnification.

Considering that ROS may play a critical role in fibrotic processes, intracellular ROS production in IM-HCF cells was detected by using a CM-H2DCFDA probe for the emission of fluorescence. TGF-β stimulation significantly increased ROS levels, as demonstrated by the augmented signal of fluorescence compared to that in control cells (not stimulated with TGF-β) (Figure 2B). ROS production was significantly reduced by the use of both A_2AR_ antagonists, Istradefylline and ZM241385, compared to with that in IM-HCFs stimulated with TGF-β alone (Figure 2C,D), thus indicating that TGF-β induced oxidative stress but the use of A_2AR_ antagonists might be protective in reducing ROS production.

### 2.3. A_2AR_ Inhibition Produces an Anti-Fibrotic Effect in Human Cardiac Fibroblasts 

**Figure 3:**
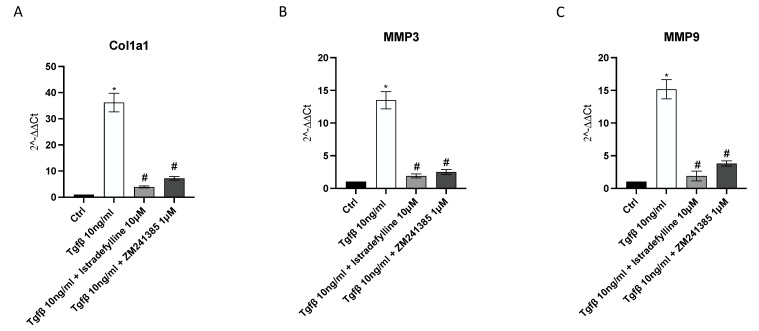
Graphs representing Col1a1 (**A**), MMP3 (**B**) and MMP9 (**C**) mRNA expression assessed via RT-PCR in IM-HCF cells. The data are expressed as the means and SDs of five experiments. * *p* < 0.05 vs. CTRL; # *p* < 0.05 vs. TGF-β.

mRNA expression of collagen1a1, MMP3 and MMP9 was studied via qPCR to investigate the anti-fibrotic effects of Istradefylline and ZM241385. TGF-β challenge caused a significant increase in Col1a1, MMP3 and MMP9 gene expression compared to that in unstimulated cells. IM-HCF cells treated with Istradefylline and ZM241385 showed a marked reduction in all investigated targets following 24 h of treatment compared to levels in the TGF-β untreated group. Treatment with Istradefylline and ZM241385 alone caused a marked reduction in Col1a1 and MMP3 gene levels, whereas it did not affect the expression of MMP9 compared to that in untreated cells (Figure 3).

**Figure 4:**
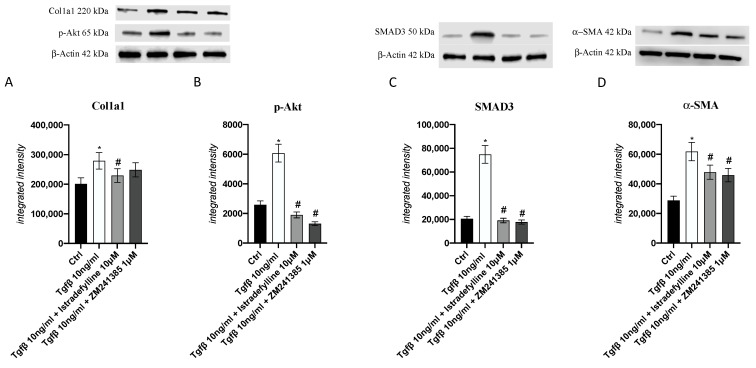
Graphs representing Col1a1 (**A**), p-Akt (**B**), SMAD3 (**C**) and α-SMA (**D**) protein expression in IM-HCF cells evaluated by Western blotting. The data are expressed as the means and SDs of five experiments. * *p* < 0.05 vs. CTRL; # *p* < 0.05 vs. TGF-β.

To better understand the A_2AR_ antagonist mode of action following application of a pro-fibrotic stimulus, collagen1a1, pAkt, SMAD3 and α-SMA protein expression was investigated; as shown in Figure 4, the protein levels of all investigated targets were increased after TGF-β challenge compared to those in untreated cells, whereas their protein expression was markedly reduced by Istradefylline or ZM241385 treatment. 

### 2.4. A_2AR_ Inhibition Modulates BGN, IL-1β and Caspase-1 Expression following TGF-β Stimulation 

**Figure 5:**
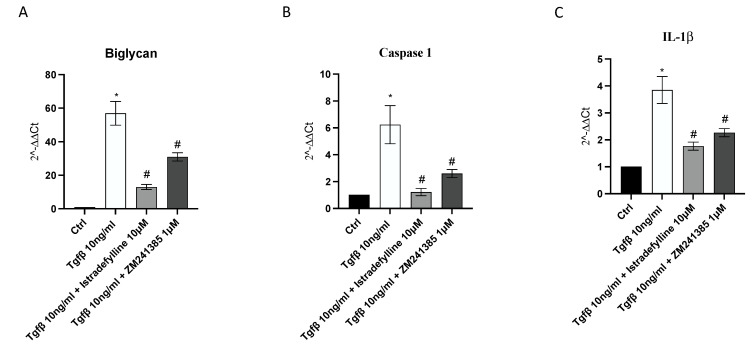
The graphs represent biglycan (**A**), caspase1 (**B**) and IL-1β (**C**) mRNA expression assessed via RT-PCR in IM-HCF cells. The data are expressed as the means and SDs of five experiments. * *p* < 0.05 vs. CTRL; # *p* < 0.05 vs. TGF-β.

BGN may trigger the inflammatory process through the activation of caspase-1 and the consequent release of IL-1β, which plays a central role both in inflammation and fibrosis; therefore, their gene expression was evaluated to confirm the anti-fibrotic mechanism of Istradefylline and ZM241385 mediated by A_2A_ receptor antagonism. A marked increase in BGN, caspase1 and IL-1β mRNA expression was observed after TGF-β stimulation; both treatments caused the significant downregulation of their expression compared to that in cells stimulated with TGF-β only. Treatment of cells with Istradefylline or ZM241385 alone did not affect BGN, caspase1 and IL-1β mRNA expression compared to that in control cells (Figure 5).

**Figure 6:**
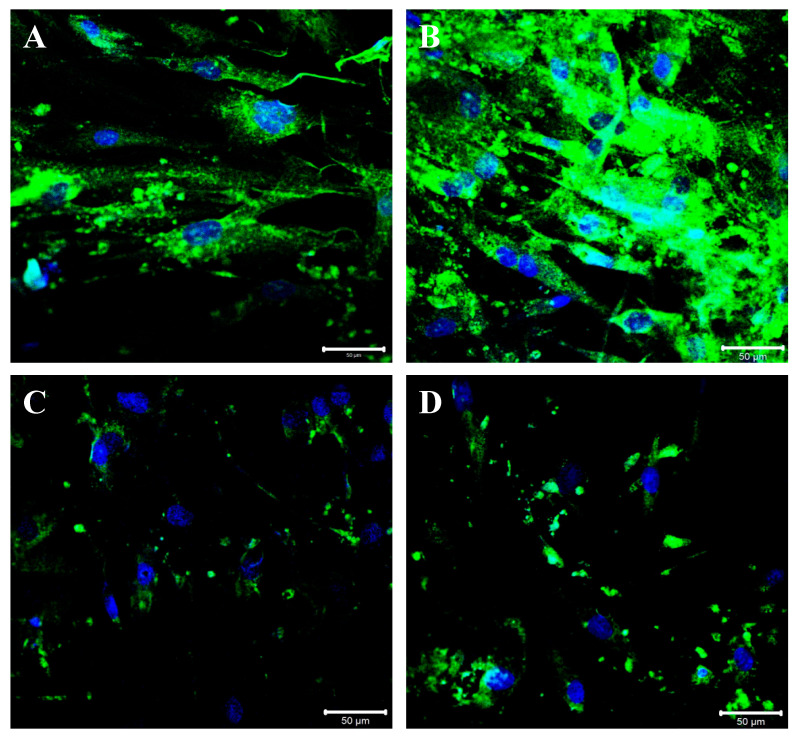
Compound panel of immunofluorescence reactions using an anti-biglycan antibody (green fluorescence). The control cells show an intense biglycan fluorescence pattern, mainly located at the perinuclear and cytoplasmic level (**A**); the cells treated with TGF-β are characterized by an increased biglycan fluorescence pattern that is distributed at the perinuclear and cytoplasmic level (**B**). The cells treated with TGF-β/Istradefylline (**C**) and TGF-β/ZM241385 (**D**) show a strong reduction in the biglycan staining pattern at the membrane and cytoplasmic level; the fluorescence pattern remains more detectable in the perinuclear region.

BGN expression modulation was also confirmed following immunofluorescence staining: an intense BGN fluorescence pattern (green channel) was detected at the perinuclear and cytoplasmic level in control cells (Figure 6A). The perinuclear and cytoplasmic localization of BGN was observed in TGF-β-stimulated cells, and the staining pattern was increased compared to that in control cells (Figure 6B). A strong reduction in the BGN fluorescence pattern may be appreciated following Istradefylline treatment compared to that in controls and the fluorescence pattern was mainly located in the perinuclear space (Figure 6C). Moreover, cells treated with ZM241385 showed a significant decrease in BGN expression, and the fluorescence pattern was mainly distributed around the nuclei (Figure 6D).

## 3. Discussion 

In the present study, the effects of two different A_2A_ receptor antagonists were evaluated in terms of the modulation of BGN expression together with other representative fibrotic markers in a TGF-β-induced model of fibrosis in human cardiac fibroblasts. 

In the context of cardiovascular system physiology, the adenosine pathway plays a key role and its deregulation and has been observed in different heart diseases [33,34]; for this reason, the modulation of adenosine receptor signaling has been proposed as a useful therapeutic tool in the cardiology field [32].

Adenosine is a purine nucleoside that exerts its physiological and pharmacological effects by engaging four types of receptors—A_1R_, A_2AR_, A_2BR_, and A_3R_. Adenosine receptors (ARs) belong to the family of G protein-coupled receptors (GPCRs), which finely regulate the cardiovascular system [34]. In particular, A_2AR_ was demonstrated to be critically involved in wound healing, and its enhanced activation in human fibroblasts was associated with increased fibrosis and collagen deposition through cyclic AMP (cAMP) and AKT activation [35]. This evidence led to the hypothesis that the modulation of A_2AR_ could be helpful in reducing the aberrant collagen deposition observed in pathological conditions characterized by fibrosis, including cardiovascular diseases.

TGF-β was used as a pro-fibrotic stimulus in human cardiac fibroblasts to provide an answer to our hypothesis, and after 24 h of incubation, ZM241385 (1 μM) and Istradefylline (10 μM), two well-known A_2AR_ antagonists, were used for an additional 24 h. The obtained data indicated that both antagonists reduced collagen1a1 production in IM-HCF cells, although a greater effect was observed following Istradefylline challenge. Uncontrolled ECM remodeling is a key pathologic feature of heart fibrosis and failure; pro-fibrotic triggers also stimulate matrix metalloproteinases, which are involved in newly formed ECM degradation, but simultaneously, activated myofibroblasts produce new ECM proteins, thus contributing to their accumulation and inducing cell disorientation and dysfunction (linked to advanced fibrosis) [36].

MMP-3 and MMP-9 are two ECM regulatory proteins released by cardiac fibroblasts and critically involved in myocardial remodeling and fibrosis [37] of which expression, as expected, was found to be increased following TGF-β stimulation and was reduced after ZM241385 and Istradefylline use, with a superimposable trend compared to that observed for Col1A1. 

The ROS released by cardiac fibroblasts in response to TGF-β exposure may induce MMP activation, which in turn regulates collagen turnover during ECM remodeling in cardiac fibroblasts; however, different processes are suppressed in altered cardiac remodeling, and oxidative stress would seem to have a critical role in the mechanisms that result in fibrosis and heart failure [38,39]. For this reason, the effects of A_2AR_ blockade were evaluated based on ROS production; increased ROS levels were detected in cells incubated with TGF-β alone, whereas they were significantly reduced after ZM241385 and Istradefylline administration. On one hand, these results are suggestive of the role of A_2AR_ role in oxidative stress and also in cardiac fibroblasts, and on the other hand, they allow us to hypothesize that the effects of both A_2AR_ antagonists could be due, at least in part, to ROS reduction. 

In this setting we also evaluated the effects of ZM241385 or Istradefylline on the expression of smad-3, of which signaling critically regulates the fibroblast phenotype and function [40,41,42,43,44], and of α-SMA, an accurate marker of activated cardiac fibroblasts, usually used to quantify fibrotic processes [42,43,44,45]. As shown during TGF-β challenge, the use of ZM241385 or Istradefylline was able to significantly decrease the expression of these molecules confirming the importance of A2_AR_ antagonism in the control of fibrosis in the heart and also modulating the switch of myofibroblasts to fibroblasts.

The proteoglycan BGN is an extracellular matrix component critically involved in collagen fibrillogenesis and adaptive remodeling after myocardial infarction, and its upregulation is responsible for impaired fibrotic scar deposition in the heart tissue [18,46]. 

As shown, BGN expression was significantly upregulated after TGF-β challenge; on the contrary, its expression was significantly reduced in cells treated with Istradefylline or ZM241385, which is an effect mediated by A_2AR_ signaling inhibition. These findings were further confirmed via immunofluorescence labeling for BGN in IM-HCF cells. As shown, after TGF-β challenge, BGN fluorescence was significantly increased, and on the contrary, the fluorescence signal decreased in Istradefylline- or ZM241385-treated cells. These new data suggest that the A_2AR_ pathway may be involved in the modulation of BGN expression and clearly show that the use of A_2AR_ antagonists could be a useful pharmacological approach in reducing the aberrant expression of such PG during the onset of fibrotic processes.

Of note, the data described so far are in contrast with those reported by Perez-Aso et al. using a murine model of fibrosis, which demonstrated that the increase in BGN expression was not prevented by ZM241385 administration [47]. These two conflicting results might be explained by the use of different experimental models; cardiac fibroblasts have a distinct pattern of gene expression compared to other fibroblasts, such as those observed in the skin [48]. Furthermore, skin fibrosis was induced via mechanical injury, and therefore, our different results could also be due to other pathways activated by TGF-β under different experimental conditions. 

BGN acts as an autonomous endogenous inducer of the inflammatory process through caspase-1 activation and the consequent release of mature IL-1β mediated by Toll-like Receptor (TLR) 2/4 [49]. The increased expression of IL-1β is a central step both during the inflammatory response and fibrotic process [50]. In this setting, previous scientific evidence has shown that A_2AR_ signaling leads to NLRP3 inflammasome activation, thus inducing caspase-1 and mature IL-1β release [51]. For this reason, we proceeded in evaluating caspase-1 and IL-1β expression following Istradefylline or ZM241385 treatment; a significant increase in the expression of both caspase-1 and IL-1β was observed in TGF-β-stimulated IM-HCFs; on the contrary, A_2AR_ blockade by ZM241385 or Istradefylline reduced the expression of both targets. This result correlates with that obtained for BGN and clearly enforces the role of A_2AR_ in NLRP3 pathway regulation [52]. 

A_2AR_ mediates collagen type 1 expression through different pathways, such as cAMP and AKT [35]; in fact, its pharmacological inhibition abrogates fibrosis in murine models of scleroderma and fibrosis by modulating these pathways [47]. Indeed, A_2AR_ inhibition, through both antagonists, also significantly reduced p-AKT expression in this in vitro TGF-β-activated experimental model; this result indicates that collagen type 1 production involves AKT, which is recruited by A_2AR_.

## 4. Materials and Methods

### 4.1. Cell Culture 

Immortalized human cardiac fibroblasts (IM-HCF) were provided by Innoprot (Derio, Spain). IM-HCF cells were cultured in Fibroblast basal Medium-2 (500 mL supplemented with 25 mL of fetal bovine serum (FBS), 5 mL of Fibroblast Growth supplement-2 and 5 mL of a penicillin/streptomycin solution (Innoprot, Derio, Spain) in a humidified incubator at 37 °C with 5% CO_2_. The culture medium was replaced every 2–3 days. Experiments were performed using fibroblast cultures between the third and the fifth passage.

### 4.2. Cell Treatments 

IM-HCF cells were cultured in six-well plates at a density of 1.5 × 10^6^ cells/well; the day after, cells were stimulated with TGF-β at the concentration of 10 ng/mL for 24 h to induce a fibrotic phenotype. Cells were treated with two different A_2A_ receptor antagonists, Istradefylline (Tocris Bioscience Bristol, Bristol, UK) and ZM241385 (Tocris Bioscience Bristol, Bristol, UK), for 24 h, at concentrations of 10 µM and 1 µM, respectively, alone or following TGF-β challenge, as previously described [53,54].

### 4.3. MTT Assay

MTT assays were carried out to assess cell viability at the end of the incubation with both TGF-β and treatments. In detail, IM-HCF cells were seeded in a 96-well plate at a density of 1 × 10^5^ cells/well and treated with TGF-β (10 ng/mL) alone and TGF-β followed by Istradefylline (10 µM) or ZM241385 (1 µM) for 24 h. Five hours before the end of the treatment, tetrazolium dye MTT 3-(4,5-dimethylthiazol-2-yl)-2,5-diphenyltetrazolium bromide (Alfa Aesar, Heysham, UK) (20 µL) was dissolved in sterile PBS and added into each well; the insoluble formazan crystals were dissolved with 200 μL/well of dimethyl sulfoxide (DMSO) following 24 h. Cell viability was measured using a VICTOR Multilabel Plate Reader (Perkin Elmer; Waltham, MA, USA) at λ 540 and 620 nm. Results are expressed as the percentage of cell viability compared to that in untreated cells. 

### 4.4. Measurement of ROS Production

Intracellular ROS production in IM-HCF cells was evaluated using the 5-(and-6)-chloromethyl-2′, 7′-dichlorodihydrofluorescein diacetate (CM-H2DCFDA) probe. In particular, after stimulation with TGF-β and treatment with the A_2A_ receptor antagonists, 5 μM of the CM-H2DCFDA probe was added into each well for 1 h at 37 °C. Cells were washed twice with sterile PBS and observed with a fluorescent microscope. The quantification of fluorescence cells was performed with ImageJ 1.53e software for Windows (Softonic, Barcelona, Spain).

### 4.5. Real-Time PCR Assay 

Total RNA was extracted from IM-HCF cells using Trizol LS Reagent Kit (Life Technologies, Monza, Italy) and quantified with a spectrophotometer (NanoDrop Lite, Thermo Fisher, Waltham, MA, USA), and then, 1 μg of total RNA was reverse transcribed using the Superscript IV RT Master Mix (Invitrogen, Carlsbad, CA, USA). Next, 1 μL of cDNA was added to the BrightGreen qPCR Master Mix (ABM, Richmond, BC, Canada) to evaluate collagen 1a1, MMP3, MMP9, caspase1, IL-1β and BGN mRNA expression. The qPCR reaction was monitored by using QuantStudio 6 Flex (Thermo fisher Scientific, Monza, MB, Italy), and the results were quantified using the 2^−ΔΔCT^ method using GAPDH as a housekeeping gene and the control group as a calibrator [55].

### 4.6. Western Blot 

After treatments, cells were collected using RIPA buffer and centrifuged, and the total protein content was quantified in cell supernatants using the Bradford method. Then, 30 μg of proteins was separated via electrophoresis on an SDS polyacrylamide gel (10%) and transferred to PVDF membranes (Amersham, Little Chalfont, UK) using a specific transfer buffer. Following washes in TBS-0.1% Tween buffer and incubation with 5% non-fat dry milk, membranes were incubated with specific primary antibodies for Col1a1, phospho-AKT (Cell Signaling, Danvers, MA, USA), SMAD3 and α-SMA (Abcam, Cambridge, UK) diluted in TBS-0.1% Tween, overnight, at 4 °C. The day after, a secondary peroxidase-conjugated goat anti-rabbit antibody (KPL, Gaithersburg, MD, USA) was used for 1 h at room temperature (RT). Images were obtained and quantified via scanning densitometry using a bio-image analysis system (C-DiGit, Li-cor, Lincoln, NE, USA). Results were expressed as relative integrated intensity using β-actin (Cell Signaling, Danvers, MA, USA) as a control for the equal loading of samples [56,57].

### 4.7. Immunofluorescence

IM-HCF cells were seeded onto a glass coverslip, fixed in 4% of paraformaldehyde (PFA) in 0.2 M phosphate buffer (pH 7.4) for 2 h at RT and then rinsed 3 times for 10 min with phosphate buffered saline (PBS). Cells were preincubated with 0.3% triton X-100 in PBS for 10 min to permeabilize the membranes and with 1% bovine serum albumin (BSA) in PBS, for 30 min at RT, in order to block nonspecific binding sites. Cells were then incubated with a rabbit polyclonal anti-biglycan antibody (1:250 dilution) (Invitrogen, Waltham, MA, USA) overnight at 4 °C. 

After rinsing with PBS, the primary antibody was detected using a FITC-conjugated IgG anti-rabbit antibody (Jackson ImmunoReseach Laboratories, Inc., West Grove, PA, USA) for 1 h at RT. Nuclei were stained with DAPI diluted to 1:1000 in PBS (Sigma Aldrich, St. Louis, MO, USA) for 10 min RT. Finally, cells were washed in PBS, and the coverslips were mounted on slides. Immunofluorescence reactions were detected and images were acquired using a Zeiss LSM 5 Duo (Carl Zeiss, Iena, Germany) confocal laser scanning microscope. All images were digitalized at the resolution of 8 bits into an array of 2048 × 2048 pixels. Optical sections of fluorescence specimens were obtained using an Argon laser (wave-length = 488 nm) at a 762 s scanning speed with up to 8 averages. Contrast and brightness were established by examining the most brightly labeled pixels and choosing the settings that allowed clear visualization of the structural details while keeping the pixel intensity at its highest (200). The parameters were chosen only based on the scans made on the control samples; once the parameters on the controls were set, the same parameters were maintained for other samples. Each image was acquired within 62 s, in order to minimize the photodegradation. Digital images were cropped, and figure montages were prepared using Adobe Photoshop 7.0 (Adobe System, Palo Alto, CA, USA).

### 4.8. Statistical Analysis

All the results are expressed as the mean ± standard deviation (SD). The reported values are the results of at least five experiments. All assays were performed in duplicate to ensure reproducibility. Different groups were analyzed via one-way ANOVA with Tukey’s post-test for intergroup comparisons. A *p*-value less than 0.05 was considered significant. Graphs were set using GraphPad Prism software (Version 8.0 for macOS, San Diego, CA, USA).

## 5. Conclusions

In conclusion, these results demonstrated that A_2AR_ blockade mediated by the use of two antagonists, ZM241385 and Istradefylline, was able to reduce the increased expression of pro-fibrotic and pro-inflammatory markers induced by TGF-β in cardiac fibroblasts. However, the role of adenosine receptors in such conditions is still conflicting, and nowadays, the “conflict” regarding the possible protective effects of A_2AR_ agonists and antagonists remains a challenge for the scientific community both with respect to the heart and other organs/tissues, probably for the management of different conditions, as well as cell types. However, our data clearly show the intracellular rearrangement of BGN at the perinuclear and cytoplasmatic level following TGF-β stimulation in cardiac fibroblasts, thus highlighting not only the important role of this PG in the transmission of pro-fibrotic signals but also that its activity may be controlled through A_2AR_ modulation. As previously mentioned, heart remodeling is a very complex process, in which different cell types and molecular pathways are engaged. For this reason, the description of merely in vitro experimental data represents the main limitation of this study, and therefore, further in vivo studies will elucidate the mechanisms ascribed to the fascinating pathways of adenosine receptors.

## Data Availability

The data presented in this study are available on request from the corresponding author.
